# Association of transcription factor 7-like 2 gene polymorphisms with breast cancer risk in northwest Chinese women

**DOI:** 10.18632/oncotarget.12591

**Published:** 2016-10-12

**Authors:** Weili Min, Xinghan Liu, Ye Lu, Zhuoqing Gong, Meng Wang, Shuai Lin, Huafeng Kang, Tianbo Jin, Xijing Wang, Xiaobin Ma, Kang Liu, Cong Dai, Yi Zheng, Shanli Li, Qingyong Ma, Zhijun Dai

**Affiliations:** ^1^ Department of Oncology, Second Affiliated Hospital of Xi'an Jiaotong University, Xi'an 710004, China; ^2^ Department of Student Affairs, Second Affiliated Hospital of Xi'an Jiaotong University, Xi'an 710004, China; ^3^ Department of Health Science Center, Xi'an Jiaotong University, Xi'an 710061, China; ^4^ Department of Hepatobiliary Surgery, First Affiliated Hospital of Xi'an Jiaotong University, Xi'an 710061, China

**Keywords:** TCF7L2, breast cancer, susceptibility

## Abstract

Genetic variations in transcription factor 7-like 2 (TCF7L2) are associated with cancer risk. This study was conducted to establish the relationship between TCF7L2 polymorphisms (rs1225404, rs7003146, and rs7903146) and clinical features and risk of breast cancer in Northwest Chinese Han women. In this study, three polymorphisms of TCF7L2 (rs1225404, rs7003146, and rs7903146) were genotyped in 458 patients with breast cancer and 500 healthy controls using the Sequenom MassARRAY-iPLEX system. We evaluated the associations between the polymorphisms and breast cancer using odds ratios (ORs) and corresponding 95% confidence intervals (95% CIs). The C allele of rs1225404 was associated with increased breast cancer risk (OR = 1.58, *P* = 0.0004, *P_C_*= 0.0012), whereas the G allele of rs7003146 was associated with decreased breast cancer risk (OR = 0.71, *P* = 0.01, *P_C_*= 0.03). Furthermore, the rs1225404 polymorphism positively correlated with negative progesterone receptor status. A positive correlation with positive estrogen receptor (ER) status was observed for the rs7003146 polymorphism. Our results suggest that TCF7L2 polymorphisms rs1225404 and rs7003146, but not rs7903146, may affect breast cancer risk in Northwest Chinese women. Additionally, the tag polymorphisms in TCF7L2 are associated with the clinical features of breast cancer, which may provide us novel insight into the pathogenesis of breast cancer.

## INTRODUCTION

Breast cancer (BC) accounts for the largest proportion of women cancer globally, and has a significant tendency to onset among younger people [1]. By estimated, BC accounted for 15% (268,600) of incidence and 7% (69,500) of mortality of women tumor in 2015 [2]. The development of BC has been shown to result from complex interactions between genetic and environmental factors.

The transcription factor 7-like 2 (TCF7L2) gene is situated on human chromosome 10q25.3 and 215.9 kb long, with 17 identified exons. It is considered to be the “sweet gene”, and is closely associated with diabetes and obesity [3]. Moreover, TCF7L2 may independently influence cancer of patients with diabetes, as the TCF7L2 gene is a key component of the Wnt/b-catenin signal pathway and exerts a vial part in the regulation of cell development and growth [4]. B-catenin binds to TCF7L2 in the nucleus, which activates its transcription factor activity. This results in the expression of some specific genes such as the cyclin D1, MCP-1, and c-myc oncogenes, which is a common feature in human cancers [5–8]. Previous studies have shown that TCF7L2 is highly expressed in hepatocellular carcinoma, breast cancer, esophageal squamous cell carcinoma, and other malignant tumors, and its expression correlates with their progress [9–13].

Single-nucleotide polymorphisms (SNPs) in the TCF7L2 gene are proposed risk factors for BC development. The rs12255372 (G/T) polymorphic variant increases the susceptibility of familial BCamong German patients, [14] and the rs7903146 (C/T) polymorphism has been correlated with BC risk in Hispanic patients [15]. However, other investigations found no association between rs12255372 and breast cancer in patients from the United States [16, 17]. In another study, the rs7003146 (G/T) polymorphism significantly correlated with reduced BC risk in Han nationality of China [18] and the rs1225404 (GA/AA genotype) may be an anti-breast cancer factor in Hispanic populations [15]. Therefore, a thorough research of genetic predisposition to BC would provide a better way to explain BC pathogenesis, generate novel diagnostic or therapeutic ways.

Pervious studies reported the inconsistent conclusions about the association between the three polymorphisms (rs1225404, rs7003146, and rs7903146) and breast cancer in different ethnicities, so we performed this study to confirm the effect of the TCF7L2 polymorphisms to BC risk, we genotyped all participants from a Chinese Han population in Northwest China for the three important SNPs, rs1225404, rs7003146, and rs7903146.

## RESULTS

### Population characteristics

BC patients and healthy participants were matched according to age and menopausal state (Table 1). The mean age of the patients and controls was 49.09 ±10.52 and 48.08 ±8.28, respectively. Patients with larger tumor size (≥ 2 cm), positive LN status, and a Scarff Bloom and Richardson (SBR) grade of 1–2 comprised 66.8%, 60.0%, and 53.1% of the total, respectively. The percentage of ER-, PR-, and HER-2-positive patients was 55.9%, 54.6%, and 27.9%, respectively.

**Table 1:** Characteristics of breast cancer patients and controls

Characteristics		Cases	Control	*P* value
Number		458	500	
Age (mean ± SD)		49.09±10.52	48.80±8.28	0.61
Menopausal status				
Premenopausal		236	236	0.38
Postmenopausal		222	264	
Body mass index (kg/m^2^)				
(mean ± SD)		23.05±2.89	22.33±2.48	0.32
Tumor size				
<2 cm		152		
≥2 cm		306		
LN metastasis				
Negative		183		
Positive		275		
Histological grade				
SBR 1-2		243		
SBR 3		215		
Venous invasion				
None–little		292		
Moderate–severe		166		
Immunohistochemistry results				
ER	−	202		
	+	256		
PR	−	208		
	+	250		
Her-2	−	330		
	+	128		

### Genotypes and alleles distributions between breast cancer individuals and healthy controls

In Table 2, we list the genotype and allele frequencies of the three TCF7L2 tag SNPs in the two groups. The results are in accordance with the Hardy-Weinberg equilibrium (HWE).

**Table 2:** Genotype and allele frequencies of TCF7L2 polymorphisms among the cases and controls and the associations with breast cancer risk

SNP	Genotype	Case	Control	P	P_C_	OR (95% CI)
rs1225404 HWE=0.85	T	751	878			1
	C	165	122	0.0004	0.0012	1.58 (1.23-2.04)
	TT	316	394			1
	CT	119	90	0.002	0.006	1.65 (1.21-2.25)
	CC	23	16	0.08	NS	1.79 (0.93-3.45)
	CT-CC	142	106	0.006	0.018	1.67 (1.25-2.24)
rs7003146 HWE=0.28	T	807	840			1
	G	109	160	0.01	0.03	0.71 (0.55-0.92)
	TT	357	355			1
	GT	93	130	0.03	0.09	0.71 (0.52-0.96)
	GG	8	15	0.15	NS	0.53 (0.22-1.27)
	GT-GG	101	145	0.01	0.03	0.69 (0.52-0.93)
rs7903146 HWE=0.54	C	857	932			1
	T	59	68	0.75	NS	0.94 (0.66-1.35)
	CC	401	435			1
	CT	55	62	0.85	NS	0.96 (0.65-1.42)
	TT	2	3	0.72	NS	0.72 (0.12-4.35)
	CT-TT	57	65	0.8	NS	0.95 (0.65-1.39)

Two-sided *x*^2^ test for the distributions of genotype and allele frequencies.

P_C_, P values after the Bonferroni correction.

NS, no significance.

As displayed in Table 2, the TCF7L2 rs1225404 polymorphism has three different genotypes: TT, CT, and CC. In the patient and control participants, the proportions of the TT, CT and CC genotype frequencies were 69%, 26%, and 5%, and 78.8%, 18%, and 3.2%, respectively. The difference in the frequency distribution of the CC and TT genotypes between the two teams was not meaningfully significant (*P* = 0.08). The rs1225404 CT genotype significantly increased BC susceptibility compared with the rs1225404 TT genotype (OR = 1.21, 95% CI = 1.21–2.25, *P* = 0.002), even after Bonferroni correction (*P_C_*= 0.006). In addition, the frequency of the rs1225404 C allele was significantly higher in BC patients than that in health (C vs. T: OR = 1.58, 95% CI = 1.23–2.04, *P* = 0.0004). The similar trend was also found even after Bonferroni correction was performed (*P_C_*= 0.0012).

We compared the GG and TT genotype frequencies of the TCF7L2 rs7003146 polymorphism in the BC group and health group and did not found any meaningful difference (*P* = 0.15). The rs7003146 T and G allele frequencies in the two groups were 82.2% and 18.0%, and 87.8% and 12.8%, respectively. We found that the GT genotype of rs7003146, and G allele specifically, had a protective effect on breast cancer risk (GT vs. TT: OR = 0.71, 95% CI = 0.52–0.96, *P* = 0.03; G vs. T: OR = 0.71, 95 % CI = 0.55–0.92, *P* = 0.01, *P_C_*= 0.03). But when the Bonferroni correction was conducted, the protective effect of GT on breast cancer risk was lost (*P_C_* = 0.09).

The TCF7L2 rs7903146 polymorphism has three genotypes: CC, CT, and TT. We compared the frequency of the CC genotype with those of the CT and TT genotypes and found no statistical difference between the patient and control groups (CT vs. CC: *P* = 0.85; TT vs. CC: *P* = 0.72). The rs7903146 C and T allele frequencies in the patient and control groups were 93.6% and 6.4%, and 93.2% and 6.8%, respectively. The difference in the two groups was not statistically significant (T vs. C: OR = 0.94, 95% CI = 0.66–1.35, *P* = 0.75).

### Relationship between rs7003146, rs1225404, and rs7903146 genotypes and the clinical pathology wof breast cancer tissues

**Figure 1:**
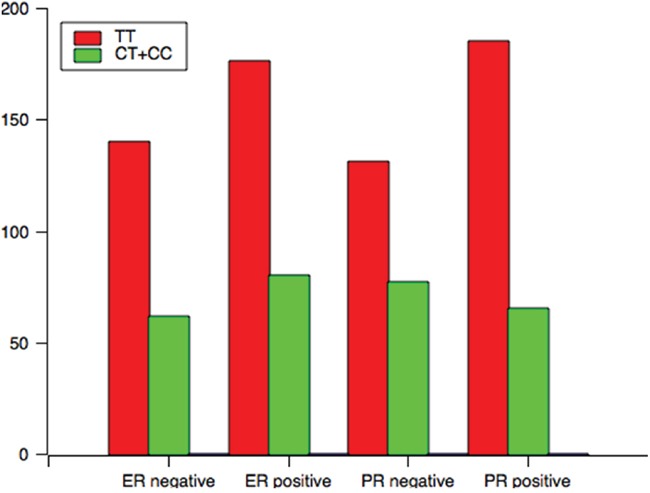
The genotype distribution of rs1225404 polymorphism among patients associated with clinical variables associated with ER and PR status

**Figure 2:**
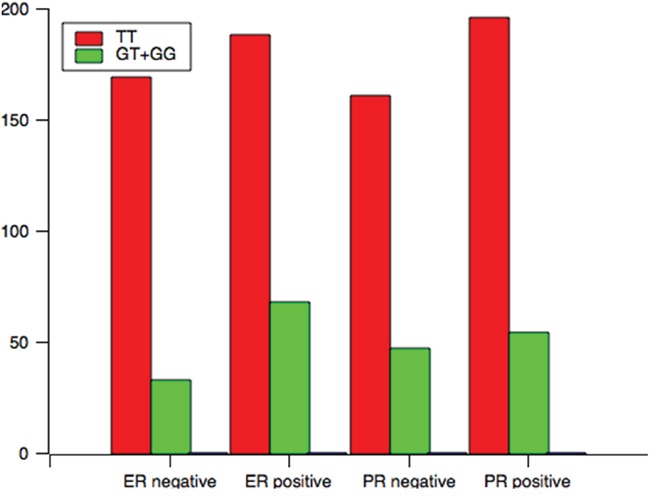
The genotype distribution of rs7003146 polymorphism among patients associated with clinical variables associated with ER and PR status

Analysis of the TCF7L2 rs1225404 polymorphism revealed a significant difference in tumor size between patients with the CT genotype, or CT and CC genotypes compared with the TT genotype, independent of other clinical and pathological data (Figure 1, CT: OR = 1.72, *P* = 0.02; CT + CC: OR = 1.71, *P* = 0.02). However, there was no difference when we performed the Bonferroni correction (P_C_ = 0.06). A relation between CT/CC genotypes and positive PR status was also observed (*P* = 0.01, OR = 0.60, *P_C_* = 0.03). GT and GG genotypes at the TCF7L2 rs7003146 locus have a correlation with positive ER status (Figure 2, OR = 1.86, 95% CI = 1.16–2.95, *P* = 0.01, *P_C_* = 0.03), compared with the TT genotype. LN involvement was significantly higher in patients with TCF7L2 rs7903146 CT and TT genotypes compared with patients with the CC genotype (OR = 2.02, *P* = 0.03). When the Boferroni correct was conducted, the significance was lost (*P_C_* = 0.09). Table 3 and Supplementary tables showed genotype and allele frequency distributions for each of the three TCF7L2 tag SNPs as well as the clinical pathology of breast cancer tissues.

**Table 3:** The associations between TCF7L2 polymorphisms and clinical characteristics of breast cancer patients

Variables	rs1225404					rs7003146				
	TT	CT+CC	P^†^	P_C_	OR (95%CI)	TT	GT+GG	P^†^	P_C_	OR (95%CI)
ER										
Negative	140	62	0.9	NS	1.03 (0.69-1.53)	169	33	0.01	0.03	1.85 (1.16-2.95)
Positive	176	80				188	68			
PR										
Negative	131	77	0.01	0.03	0.60 (0.40-0.89)	161	47	0.8	NS	0.94 (0.61-1.47)
Positive	185	65								

Two-sided χ^2^ test for the distributions of genotype frequencies.

P^†^: Adjusted for tumor size, lymph node involvement, histological grade, venous invasion, ER, PR, HER-2, and Ki67 status.

P_C_: P values after the Bonferroni correction.

ER: Estrogen receptor; PR: Progesterone receptor.

### Relationships between rs1225404, rs7003146, and rs7903146 haplotypes and breast cancer susceptibility

The three polymorphisms were in LD, and then we conducted the haplotype analysis. As shown in Table 4, we choose the most frequent haplotype (T_rs1225404_T_rs7003146_C_rs7903146_) in the controls as the reference and compared the other haplotypes with T_rs1225404_T_rs7003146_C_rs7903146_. No significant relationships were observed between all haplotypes and BC risk (*P* >0.05), which indicated the three polymorphisms may affect the breast cancer risk in separate ways.

**Table 4:** Relationships between rs1225404, rs7003146, and rs7903146 haplotypes and breast cancer risk

Haplotypes			Cases (N=916)	Controls (N=1000)	OR (95% CI)	*P*
rs1225404	rs7003146	rs7903146				
T	T	C	642	713		
T	T	T	30	37	0.90 (0.55-1.48)	0.71
C	T	T	27	43	0.70 (0.43-1.14)	0.18
C	T	C	192	188	1.13 (0.90-1.42)	0.30
CGC, TGT, CGT			25	19	1.46 (0.80-2.68)	0.22

## DISCUSSION

In this study, we found that the C allele and CT genotype of the rs1225404 polymorphism were increased risk factors during breast cancer progression (OR = 1.21, *P* = 0.002). The frequencies of rs1225404 genotypes were positively correlated with positive PR status (*P*< 0.05), indicating that genetic variations at the locus may influence the prognosis of the breast cancer patients. In contrast, Connor's study surmised that the CC genotype of rs1225404 was inversely related with breast cancer susceptibility [15]. The results of our study had some different from others' researches, which probably because of different races, sample size or pathological type of tumor.

The TCF7L2 rs7003146 GT genotype frequency in the patients was significantly different from that in the controls (OR = 0.71, *P* = 0.03). Therefore, consistent with the research of Wei and Tang, [8, 18] the rs7003146 GT genotype may reduce breast cancer risk. Further stratified analysis showed that rs7003146 positively correlated with positive ER status, which is considered a predictive marker of effect of endocrine therapy. Thus, the rs7003146 polymorphism may contribute to endocrine treatment of breast cancer and related to protective factors for breast cancer.

The rs7903146 polymorphism is located in an intronic region of TCF7L2 and known to be related toan increased type 2 diabetes risk. The rs7903146 polymorphism also has high linkage disequilibrium with rs12255372 and microsatellite DG10S478. [3] Previous studies have shown that the TCF7L2 rs7903146 polymorphism may increase breast cancer [8, 15, 17], lung cancer [19], and colorectal cancer [19] risk. However, our results suggest that the TCF7L2 rs7903146 polymorphism might have no association with breast cancer risk. Furthermore, in a stratified analysis, rs7903146 (T) is positively correlated with BMI, which is one of the risk factors for breast cancer. rs7903146 (T) is also associated with LN status, which is reported by Naidu [20]. Patients with positive LN status had higher mortality. These results suggest that, rather than contributing to tumor initiation, the variant of rs7903146 may increase proliferation of transformed cells and increase tumor metastatic potential and/or decrease apoptosis in malignant cells.

The molecular mechanism of TCF7L2 gene polymorphisms and altered cancer risk remains unclear. Chen *et al.* [21] described that the gene-gene interactions between TCF7L2 rs7094463, rs10749127, and rs11196224 played an important role in the disease recurrence in prostate cancer patients suffering from radical prostatectomy. ENCODE data showed that rs7094463 is situated in a locus that has a promoter histone modification patterns and within the II binding region of RNA polymerase; rs10749127 and rs11196224 polymorphisms were consistent withopen chromatin regions, which probably correspond to TCF7L2 enhancers. Additionally, we have found the alleles of the three polymorphisms were in linkage disequilibrium and these haplotypes correlate with increased breast cancer risk [22].

The three SNPs analyzed in our study are located within an intron, so these TCF7L2 gene polymorphisms may be affecting gene-gene interactions. We should figure out if these SNPs alter the expression of TCF7L2 and how it happens in a give tissue in the future. However, several groups have made great efforts to explain the underlying effect of TCF7L2 in breast cancer.

In summary, we found that TCF7L2 SNPs (rs1225404 and rs7003146) might be associated with breast cancer risk in Northwest Chinese Han populations. Combined with previous studies, our data indicate that such polymorphisms could be a potential candidate for breast cancer susceptibility. Our case-control study helps to understand the possible pathogenesis breast cancer. However, TCF7L2 has multiple polymorphic sites, and their associations with breast cancer are uncertain. Further studies, with larger sample sizes, are needed to validate our conclusionsand the role of these variants in breast carcinogenesis.

## MATERIALS AND METHODS

### Study population

A case-control study was performed with 458 breast cancer patients randomly selected from the Second Affiliated Hospital of Xi'an Jiaotong University between January 2013 and March 2014. All patients, who were Han Chinese, had undergone modified radical mastectomy or radical mastectomy reservations and had complete clinical pathological data. The tumor diameter range was 1.6–4.5 cm (mean diameter, 2.2 cm). Pathological analysis of the samples confirmed that patients had not been receiving treatment (neoadjuvant chemotherapy or endocrine therapy) before surgery, and all of the histological classifications were referenced to NCCN 2013 Guidelines. Menopausal status was defined as the date of last menses followed by 12 months without menses. The clinico-pathological variables and prognostic factors, including tumor size, histology, axillary lymph node (LN) involvement, hormone receptor (including estrogen receptor (ER) and progesterone receptor (PR)) status, and human epidermal growth factor receptor (HER2) status, were obtained from medical records. Histological determination, including tumor type and disease stage, was performed according to the World Health Organization criteria and the TNM classification system, respectively.

The control group was comprised of 500 unrelated, sex-, age-, and ethnicity-matched healthy individuals from the same hospital. Control subjects had no known medical illnesses or hereditary disorders and were not taking any medications. Written informed consent was obtained from the two groups at the time of recruitment. This study was approved by the Institutional Review Board of Xi'an Jiaotong University (Xi'an, China).

### Genotyping assay

Limosis venous blood (5 ml) was extracted from each subject and stored in EDTA anticoagulant tubes at -80 °C. Whole-blood genomic DNA was extracted using the salting-out technique. DNA concentrations were measured by spectrophotometry

(DU530 UV/VIS spectrophotometer, Beckman Instruments, Fullerton, CA, USA). Three tag SNPs (rs1225404, rs7003146, and rs7903146) were selected for analysis. Data from HapMap (http://www.hapmap.org) indicted that these tag SNPs capture the majority of the known common variation in TCF7L2 within the Chinese Han population. Sequenom MassARRAY Assay Design 3.0 software was used to design a Multiplexed SNP MassEXTEND assay. SNP genotyping was performed using Sequenom MassARRAY RS1000 according to manufacturer's recommendations. The primers used for each SNP are listed in Table 5. Sequenom Typer 4.0 Software was used for data management and analysis.

**Table 5:** Primers used for this study

SNP_ID	Physical position	Location	1st-PCRP	2nd-PCRP	UEP_SEQ
rs1225404	113154906	Intron region	ACGTTGGATGTTCAGT GCTGCGGTTCTTAG	ACGTTGGATGACACT CACACTCACGCCTTC	CACGCCTTCCTTTTATG
rs7003146	35884126	Intron region	ACGTTGGATGTCCTGG ATTCACGCCATACT	ACGTTGGATGCCCCGT CTCTACTAAAAAAAC	GCAGGCGCCTGTAGTC
rs7903146	112998590	Intron region	ACGTTGGATGAACTAA GGGTGCCTCATACG	ACGTTGGATGTCTCTG CCTCAAAACCTAGC	AGAGCTAAGCAC TTTTTAGATA

### Statistical analysis

Statistical analyses were performed with SPSS 18.0 for Windows (PASW Statistics, SPSS Inc., Chicago, IL). All tests were two-sided and statistical significance was set at *P*< 0.05. Bonferroni's correction was used for multiple comparisons. The frequency of each SNP was tested in control subjects to determine if they depart from HWE. We firstly performed the normality tests for age and body mass index (BMI) at diagnosis between case and control groups, and the results showed they all subjected to normal distribution. Then the student's *t*-test was used to determine the differences in age and BMI between the two groups. Chi-square (Pearson's χ^2^) test or Fisher's exact test was used, where appropriate, to calculate the *P* values and corresponding odds ratios (OR) with 95% confidence intervals (CI), in order to determine the associations between genotypes and breast cancer risk or clinical variables. We conducted the haplotype analysis under the generalized linear models by using the software PHASE.

## SUPPLEMENTARY MATERIALS TABLES


