# Supplementary material for: Association of transcription factor 7-like 2 gene polymorphisms with breast cancer risk in northwest Chinese women
Source: Oncotarget. 2016 Oct 12;7(47):77175–82. doi: 10.18632/oncotarget.12591 (PMC5363578; doi:10.18632/oncotarget.12591)
Supplement: Supplementary file 1 [file oncotarget-07-77175-s001.pdf]

Association of transcription factor 7-like 2 gene polymorphisms with breast cancer risk in northwest Chinese women

SUPPLEMENTARY TABLES

Supplementary Table S1: The associations between TCF7L2 rs1225404 polymorphism and clinical characteristics of breast cancer patients

Variables	TT	CT+CC	P†	Pc	OR (95% CI)
Age					
<50	177	83	0.63	NS	0.91 (0.61-1.35)
≥50	139	59			
Menopausal status					
Pre-	163	73	0.97	NS	1.0 (0.68-1.50)
Post-	153	69			
Tumor size					
<2 cm	116	36	0.02	0.06	1.71 (1.10-2.61)
≥2 cm	200	106			
LN metastasis					
Negative	120	63	0.2	NS	0.79 (0.51-1.15)
Positive	196	79			
Histological grade					
SBR 1-2	165	78	0.59	NS	0.90 (0.60-1.33)
SBR 3	151	64			
Venous invasion					
None–little	208	85	0.23	NS	1.28 (0.85-1.92)
Moderate–severe	109	57			
ER					
Negative	140	62	0.9	NS	1.03 (0.69-1.53)
Positive	176	80			
HER-2					
Negative	231	99	0.45	NS	1.18 (0.76-1.83)
Positive	85	43			

Two-sided χ^2 test for the distributions of genotype frequencies.
P† Adjusted for tumor size, lymph node involvement, histological grade, venous invasion, ER, PR, HER-2, and Ki67 status.
P_C: After Bonferroni correction.
LN: Axillary lymph node; SBR: Scarff, Bloom and Richardson; tumor grade (1-2 vs. 3); ER: Estrogen receptor; PR:≈Progesterone receptor; HER-2: human epidermal growth factor receptor 2.

Supplementary Table S2: The associations between TCF7L2 rs7003146 polymorphism and clinical characteristics of breast cancer patients

Variables	TT	GT+GG	P†	Pc	OR (95% CI)
Age					
<50	209	51	0.15	NS	1.38 (0.89-1.56)
≥50	148	50			
Menopausal status					
Pre-	185	51	0.81	NS	1.05 (0.68-1.64)
Post-	172	50			
Tumor size					
<2 cm	123	29	0.28	NS	1.31 (0.81-2.12)
≥2 cm	234	72			
LN metastasis					
Negative	143	40	0.94	NS	1.02 (0.65-1.60)
Positive	214	61			
Histological grade					
SBR 1-2	190	53	0.89	NS	1.03 (0.66-1.60)
SBR 3	167	48			
Venous invasion					
None–little	227	65	0.89	NS	0.97 (0.61-1.53)
Moderate–severe	130	36			
HER-2					
Negative	255	75	0.58	NS	0.87 (0.53-1.43)
Positive	102	26			

Two-sided χ^2 test for the distributions of genotype frequencies.
P† Adjusted for tumor size, lymph node involvement, histological grade, venous invasion, ER, PR, HER-2, and Ki67 status.
P_C: After Bonferroni correction.
LN: Axillary lymph node; SBR: Scarff, Bloom and Richardson; tumor grade (1-2 vs. 3); ER: Estrogen receptor; PR: Progesterone receptor; HER-2: human epidermal growth factor receptor 2.

Supplementary Table S3: The associations between TCF7L2 rs7903146 polymorphism and clinical characteristics of breast cancer patients

Variables	CC	CT+TT	P†	Pc	OR (95% CI)
Age					
<50	226	34	0.63	NS	0.87 (0.50-1.54)
≥50	175	23			
Menopausal status					
Pre-	207	29	0.92	NS	1.03 (0.59-1.78)
Post-	194	28			
Tumor size					
<2 cm	127	25	0.07	NS	0.59 (0.341.04)
≥2 cm	274	32			
LN metastasis					
Negative	168	15	0.03	0.09	2.02 (1.08-3.76)
Positive	233	42			
Histological grade					
SBR 1-2	217	26	0.23	NS	1.41 (0.81-2.45)
SBR 3	184	31			
Venous invasion					
None–little	174	28	0.42	NS	0.94 (0.46-1.38)
Moderate–severe	227	29			
ER					
Negative	174	28	0.42	NS	0.94 (0.46-1.38)
Positive	227	29			
PR					
Negative	181	27	0.75	NS	0.91 (0.52-1.59)
Positive	220	30			
HER-2					
Negative	287	43	0.54	NS	0.82 (0.43-1.56)
Positive	114	14			

Two-sided χ^2 test for the distributions of genotype frequencies.
P† Adjusted for tumor size, lymph node involvement, histological grade, venous invasion, ER, PR, HER-2, and Ki67 status.
P_C: After Bonferroni correction.
LN: Axillary lymph node; SBR: Scarff, Bloom and Richardson; tumor grade (1-2 vs. 3); ER: Estrogen receptor;
PR: Progesterone receptor; HER-2: human epidermal growth factor receptor 2.
